# Development of a portable device to quantify hepatic steatosis in potential donor livers

**DOI:** 10.3389/frtra.2023.1206085

**Published:** 2023-06-23

**Authors:** Mac Klinkachorn, Christian Tsoi-A-Sue, Raja R. Narayan, Haaris Kadri, Taylor Tam, Marc L. Melcher

**Affiliations:** ^1^ Department of Engineering, Stanford University, Stanford, CA, United States; ^2^ Department of Surgery, Stanford University, Stanford, CA, United States; ^3^ Department of Surgery, Mass General, Boston MA, United States; ^4^ Menlo School, Menlo Park, CA, United States

**Keywords:** artificial intelligence, liver transplant, organ donor, biopsy, organ assessment

## Abstract

An accurate estimation of liver fat content is necessary to predict how a donated liver will function after transplantation. Currently, a pathologist needs to be available at all hours of the day, even at remote hospitals, when an organ donor is procured. Even among expert pathologists, the estimation of liver fat content is operator-dependent. Here we describe the development of a low-cost, end-to-end artificial intelligence platform to evaluate liver fat content on a donor liver biopsy slide in real-time. The hardware includes a high-resolution camera, display, and GPU to acquire and process donor liver biopsy slides. A deep learning model was trained to label and quantify fat globules in liver tissue. The algorithm was deployed on the device to enable real-time quantification and characterization of fat content for transplant decision-making. This information is displayed on the device and can also be sent to a cloud platform for further analysis.

## Introduction

Thousands of patients die every year from the shortage of donor livers for transplantation (1). Therefore, transplant surgeons seek to expand the criteria and safe use of potentially transplantable livers. Livers with high fat content or steatosis are thought to function poorly following transplantation, increasing the risk for early graft dysfunction, need for re-transplantation, or death (2, 3). However, some have argued that these organs are transplantable especially if appropriate recipients are chosen (4). Recent reports raise concerns that manual fat scores between different pathologists can vary (5–7). More accurate estimates may enable the use of more livers. About 33% of livers considered for transplantation are biopsied as part of the evaluation process (8). Often, a community pathologist is called in to evaluate the liver tissues, night or day, wherever the liver donor is located. However, this process takes time and studies have shown significantly different fat scores reported between different pathologists (5) Recognizing this problem, several groups have sought to automate steatosis scoring (6, 7, 9, 10).

Previously, we detailed the development of a machine-learning algorithm to label fat globules with high accuracy by leveraging pre-trained neural networks built on a labeled database of donor liver slides (11, 12). The analysis of these data resided on the cloud posing practical limitations when considering their implementation in a clinical setting. First, cloud analysis depends on secure access to the internet which may not be readily accessible in remote community hospitals where donors may become available. Second, waiting for the transplant surgical team to retrieve the donor liver biopsy for analysis at the transplanting center, can delay the determination of whether the organ is safe for use and prolong cold ischemia time. Lastly, reliance on the cloud is associated with the risk of private patient data exposure while transferring health information for central analysis. The use of a point-of-care device enables the private analysis to be de-identified and/or deleted without the risks of exposure related to the cloud.

Therefore, we propose the development of an end-to-end device that leverages an artificial intelligence (AI)-based algorithm, a graphics processing unit (GPU), and a high-definition camera to detect percent steatosis in livers of patients with high precision and accuracy. The device is portable, computationally efficient, independent of internet access, and of low cost.

## Results

### Software for AI-based steatosis detection at the point-of-care

**Figure 1:**
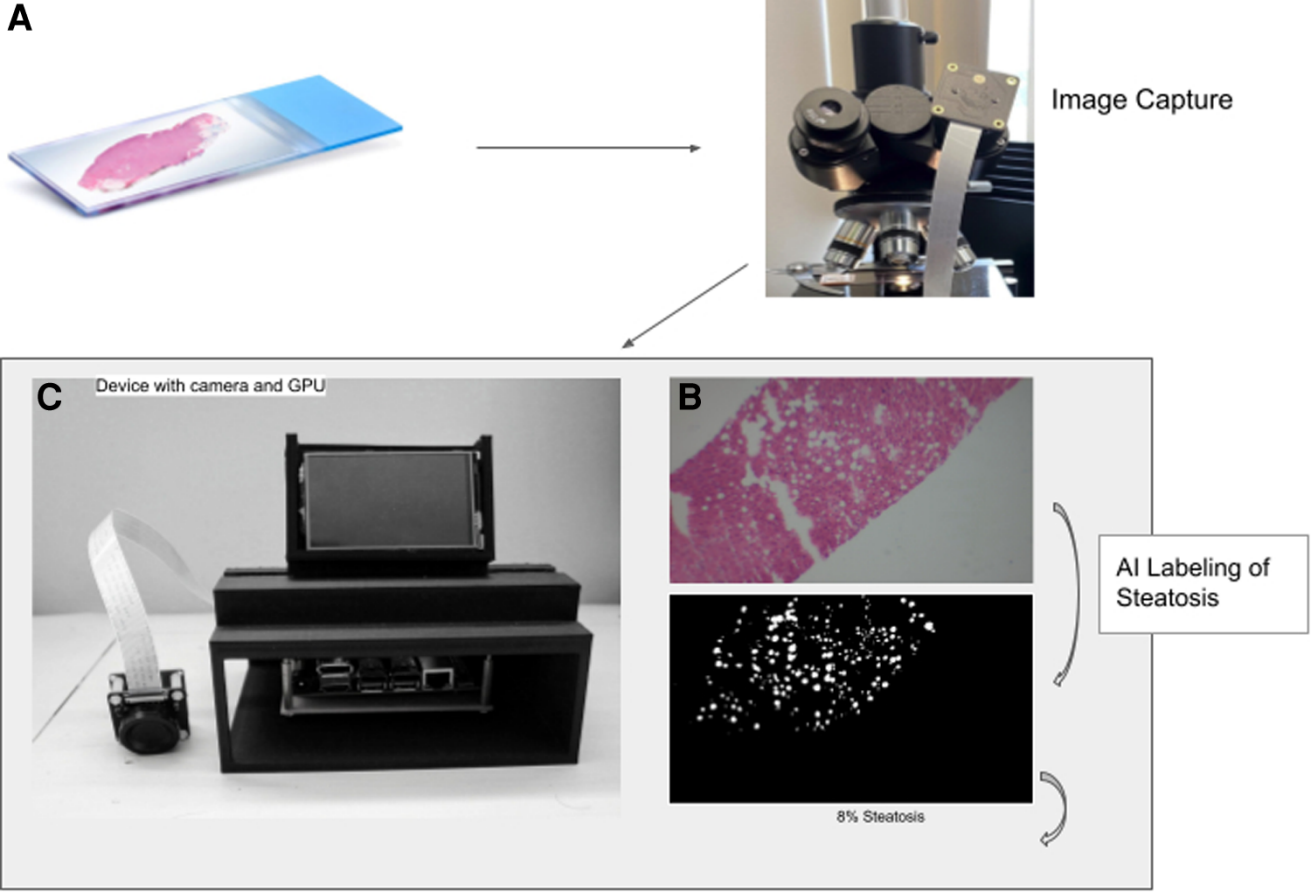
Workflow to calculate percent steatosis. The slide image (**A**) is captured (**B**) and tiled before analysis by the trained U-NET model using the GPU on the device (**C**). The algorithm generates a mask that labels fat vacuoles as white and all else as black (**D**).

There are several challenges in developing a machine learning algorithm that runs on a device. Since the algorithm for segmenting fat cells usually requires significant computational resources and memory, we use model compression techniques to transfer the machine learning model to the Nvidia Jetson Nano device (Figure 1A). We also utilize the GPU computing power to improve the latency of slide analysis and inference to allow almost real-time results.

### Hardware assembly

To take pictures directly through the microscopes’ eyepiece we mounted an IMX477—IR Cut Arducam camera module (12.3 megapixels) with low-light sensor capabilities to a 3D printed adapter. The adapter is friction fitted to the microscope eyepiece of 38 mm in diameter (Figure 1B). The camera driver has auto-focus and can adjust to low light capabilities. Less expensive cameras without low light capability did not take adequate photos. The IMX camera module connects directly to the Jetson Nano (Nvidia) via the CSI-2 port.

The Jetson Nano was mounted on a custom-designed 3D-printed case made of polylactic acid. The case has multiple open sides for easy access to ports. On the top is a vent hole designed to allow optimal airflow for the heat sync. In front of the vent is a display mount which allows for a liquid crystal display to be easily attachable and detachable (Figure 1). With the display mounted, the device is 24 cm high, 16.2 cm wide, and 14.5 cm deep.

### Device software

A Python script provides a graphical user interface to access the camera capture function natively. To reduce strain on the CPU, the resolution was set to 1080 × 720 p and the frame rate to 60 fps. Using the script, a user can trigger the device to acquire an image through the microscope and store it in the internal memory where it can be accessed for analysis.

The U-net network that we use to detect liver fat content accepts 256*256 pixel tile input (12). Therefore, we developed a script to tile the image captured from the sensor into multiple 256*256 tiles. The tiles are then passed into the neural network to create a steatosis mask. The network analyzes each pixel in the tiles to identify whether it represents a fat pixel. By programmatically counting all fat vacuoles and using a filter to remove the background, the device can estimate the percent steatosis in the liver tiles. The system then computes the average steatosis across the images that were sampled from the slides.

### Comparison between cloud platform and end-to-end device

**Figure 2:**
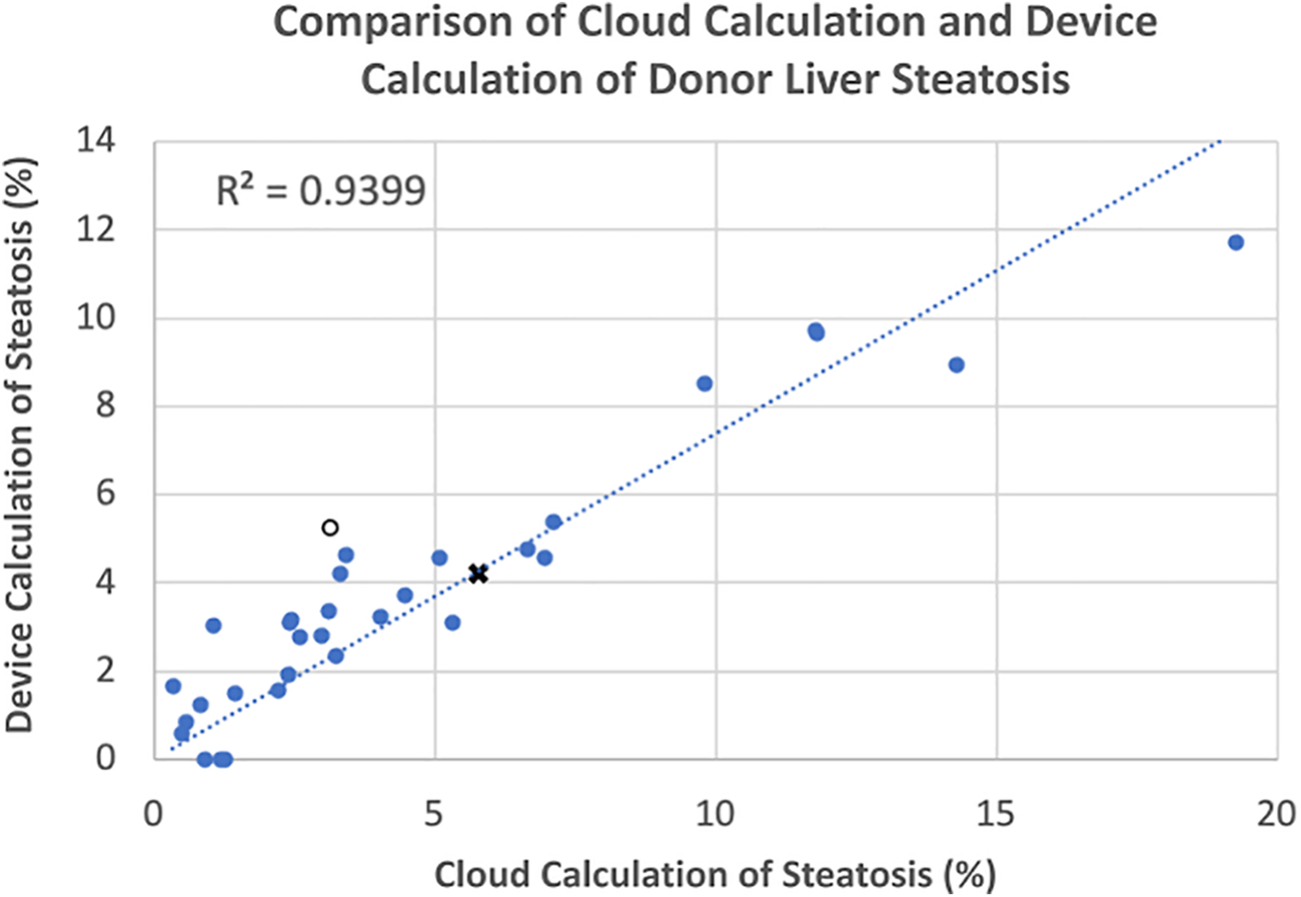
Correlation of cloud and device scored steatosis. With trend line intercept set at 0,0, a strong correlation (*r* = 0.9339) was noted with a few outliers that were subsequently inspected. Open circle, ○, point corresponds to Slide #20; X-point corresponds to Slide #26 (see Figure 3).

**Figure 3:**
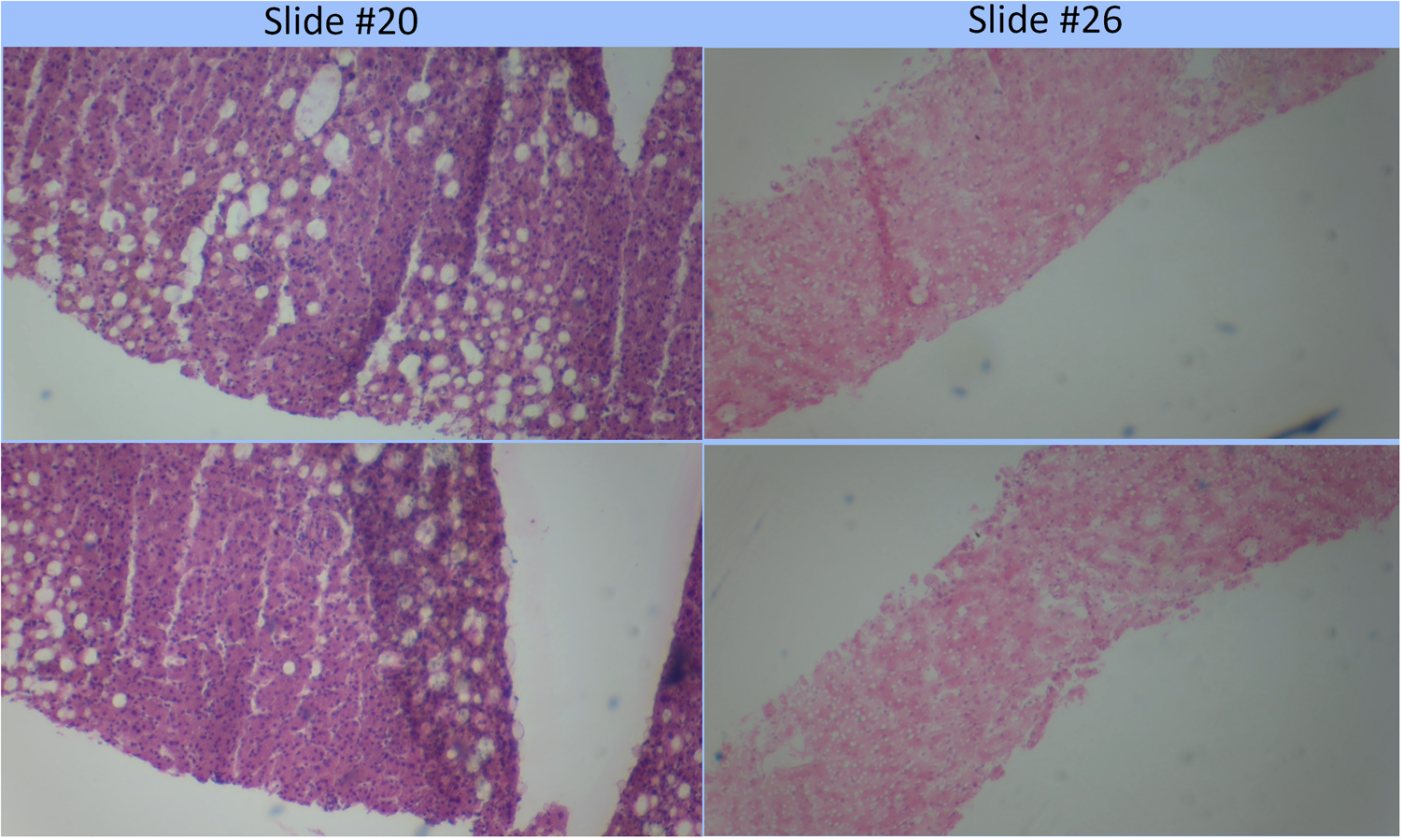
Comparison of poorly correlating slides (#20) to strongly correlating slide (#26). Poorly correlating slide #20 appears to have a more heterogeneous distribution of fat globules than the strongly correlating slide #26.

The assessment of the steatosis on 33 slides by the device was compared to the whole slide assessment using the same algorithm on a cloud-based platform previously described (12). There was a strong positive correlation (*r* = 0.9399) between the two techniques. Figure 2 shows a plot of cloud-based steatosis scores against device-derived scores. When slides that were particularly divergent between the two techniques (Slide 20) were compared to less divergent slides (Slide 26), the former had patches of steatosis rather than the uniform distribution seen on the latter (Figure 3).

## Discussion

Currently, the assessment of liver biopsies for steatosis can be variable and even difficult to obtain outside of dedicated liver transplant centers. Therefore, we developed this prototype stand-alone device capable of acquiring images of liver biopsies from a microscope, processing them, analyzing them, and measuring the percent steatosis. We hope that these assessments will facilitate the evaluation of donated livers for transplantation to reduce the number discarded and optimally match them with appropriate recipients. The device relies on the AI algorithm our group previously trained on a Google Cloud Platform to label fat vacuoles in a liver specimen. Fortunately, our previous work had shown that the algorithm was very good at recognizing artifacts in the image caused by tears in the tissue and not scoring these as steatosis (12).

While the steatosis correlation between the device and the cloud was high, it was not perfect. Despite the device capturing three images from each slide, steatosis percent varied considerably on several slides. Therefore, three images may not be enough to score steatosis reproducibly. Future iterations of this algorithm should include an assessment of the image and biopsy quality to identify images out of focus or with too many artifacts rendering the image unusable.

This end-to-end device can be used at a donor hospital to obtain images from their microscope and assess the steatosis of a donor liver without a digital scanner or a connection to the internet. The device is powerful enough that images could be analyzed on the device without needing to load large files to a cloud platform. Keeping the data on the device also reduces the danger of sending protected personal health information to the cloud. The biopsy data would not be permanently stored on the device and can be erased as soon as a result is given. The results are obtained more rapidly and reliably by using the device. Since the slides are relatively large, it can take a substantial amount of time to transfer over the network with limited bandwidth. In the case of the device, the image acquisition and analysis are done on the device using a GPU; therefore, the analysis can be completed within a few minutes.

To access the quality of a donated liver, a transplant team considers multiple donor variables including age, medical history, cause of death, and laboratory values. After the organ is provisionally accepted, a procurement team is dispatched to the hospital in which the donor candidate is located. In many circumstances, the potential donor may be at a remote community hospital that does not have an experienced, on-call, liver pathologist, who could readily screen for liver steatosis, or there may be an extended delay in bringing in an on-call pathologist to review a donor liver biopsy in the middle of the night. Moreover, review of fat globules in the community hospital setting is uncommon on hematoxylin and eosin-stained frozen biopsy slides as other stains that take days to process are the preferred modality for non-urgent clinical circumstances. In some cases, a screenshot of the donor liver biopsy slide through the microscope may be crudely sent to the supervising transplant surgeon who can review the image to visually estimate the degree of fat involvement before approving liver procurement to begin.

A point-of-care device offers several advantages over a cloud-dependent platform for donor liver biopsy analysis. First, a remote community hospital may lack the internet access and computing power necessary to utilize a robust cloud-dependent platform. Second, a point-of-care device can quickly define the degree of fat involvement without requiring a pathologist to arrive, often, in the middle of the night to review the slide before permitting transplantation. Before deciding not to use a liver solely based on the assessment provided by the device, we would recommend having a pathologist examine the slides to prevent the unnecessary discarding of livers. Third, the use of a such device for real-time, rapid evaluation of biopsies could also be advantageous in assessing the impact of machine perfusion on reducing fat content in donated livers intended for transplantation. Finally, the use of a closed system device, disconnected from the internet, reduces the risk of private health information exposure that may occur during the transference of data onto a cloud-based central system. Logistically, a device available in real-time can streamline the transplant decision-making process to limit the aforementioned barriers to transplantation.

This device is still a prototype. As such, there are several improvements to be made. Despite being relatively small, it is clunky and will benefit from useability studies to improve the design. The microscope adapter will need to come in different sizes to accommodate different lab microscopes. Currently, the use of this device still requires the slides to be prepared and therefore is not completely independent of local hospital support. Additional work needs to be done to characterize macro- versus microsteatosis and its impact on outcomes. Initial work suggested a distribution of vacuole sizes rather than two distinct populations. Biopsy characteristics such as fibrosis and inflammation are not characterized by the device and should be trained into future versions of the algorithm. In addition, it is important to acknowledge competing technologies based on increasingly powerful smartphones and access to cloud computing (9). Machine perfusion pumps may also mitigate concerns about prolonged cold ischemia times.

## Methods

### Hardware components

The components of this device include the Jetson Nano™ (Nvidia) with a graphic processing unit (GPU), a Waveshare HQ Camera with a 12.3MP IMX477High Sensitivity, a 7inch IPS capacitive touch display, ribbon connectors, a power supply, and an HDMI cable.

### Software components

We develop our platform on the native Ubuntu™ 18.04 which is the operating system on the Jetson Nano. Scripts were written in Python^TM^, with the help of the following libraries, Tensor Flow™ for machine learning and deep learning inference, OpenCV™ for image analysis, and Argus API for image ingestion.

### Liver steatosis detection algorithm

Previously, a U-net network has been pre-trained on a cell segmentation task capable of segmenting fat vacuoles with high accuracy (12). Utilizing an established U-net platform to detect the fat content in the liver tissues, the algorithm was able to assess every pixel on a liver donor biopsy slide to determine whether they represent fat vacuoles or normal liver cells.

### Imaging and data collection

Three images of each slide were acquired by the device. The physician can adjust the microscope to different areas of the slides and use the touchscreen on the device to collect the sample images on the area of interest. After the image acquisition, several filters are applied to confirm that the picture is stable and remove any background noise from the data collection process. The images are then processed into 256 × 256-pixel tiles to be analyzed by the AI algorithm. Within these tiles, the AI algorithm labels each pixel as a “one” for the presence of steatosis or a “zero” for the absence of steatosis to create the mask.

### Evaluation metrics

The capability of device analysis to the cloud analysis was done by plotting the two modalities against each other.

## Data Availability

The raw data supporting the conclusions of this article will be made available by the authors, without undue reservation.
